# Current biomarker development in myotonic dystrophies

**DOI:** 10.1007/s00415-026-13888-w

**Published:** 2026-06-14

**Authors:** Malin Larsen, Daniel H. Mendelsohn, Felix Kleefeld, Peter Meinke, Benedikt Schoser

**Affiliations:** 1 Department of Neurology, Friedrich-Baur-Institute, LMU Clinics, Munich, Germany; 2 Department of Neurology, Berufsgenossenschaftliches University Hospital Bergmannsheil Bochum, Bochum, Germany; 3 Heimer Institute for Muscle Research, Berufsgenossenschaftliches University Hospital Bergmannsheil Bochum, Bochum, Germany; 4 Department of Neurology, Charité Universitätsmedizin Berlin, Berlin, Germany

**Keywords:** Myotonic dystrophy, Biomarkers, Alternative splicing, Muscle-derived biomarkers, Soluble biomarkers, Imaging biomarkers

## Abstract

Myotonic dystrophies (DM) are autosomal dominant, multisystemic disorders characterized by myotonia and progressive muscle weakness. Extramuscular multisystem symptoms include involvement of the respiratory, cardiac and central nervous systems, and endocrine and autoimmune alterations. While there is significant overlap in key symptoms due to a shared molecular backbone of alternative splicing, DM1 and DM2 differ in genetic origin, disease progression, and aspects of molecular pathology. Currently, DM management focuses on symptoms, and the wide clinical variation leads to long diagnostic delay. This highlights the need for reliable biomarkers to aid diagnosis, prognosis, and monitoring. As the understanding of molecular mechanisms in DM improves, new therapies are rapidly emerging, further emphasizing the need for reliable biomarkers to assess short- and long-term treatment efficacy. This review covers current biomarker research, including imaging-based, muscle-derived, and soluble liquid biopsy approaches. While established DM biomarkers are limited, with research disproportionately focused on DM1, multiple markers of alternative splicing pathology show promise. However, their reliance on invasive sampling restricts cohort size and longitudinal assessment. Consequently, efforts are shifting toward developing minimally invasive biomarkers. While some soluble markers, like muscle-specific circulating microRNAs, correlate with clinical measures, inconsistent detection, and limited evaluation of disease specificity point to the need for further research in larger, more diverse longitudinal cohorts. Future progress will depend on validating existing candidates, discovering new biomarkers addressing these limitations, and reducing the imbalance between DM1- and DM2-focused research to advance DM diagnosis, management, and therapy development.

## Introduction and clinical overview

Myotonic dystrophies (DM) are autosomal dominant, multisystemic disorders predominantly characterized by myotonia and progressive muscle weakness. Distinguished by diverging genetic origins, there are two forms of DM. Myotonic dystrophy type 1 (DM1), caused by a CTG repeat expansion in the 3′ untranslated region of the dystrophia myotonica protein kinase gene (DMPK) [1, 2], and myotonic dystrophy type 2 (DM2) which stems from a CCTG expansion within a complex repeat motif in intron 1 of CNPB, the gene encoding the CCHC-type zinc finger nucleic acid-binding protein [3, 4]. DM1 is the most common cause of adult muscular dystrophy, with recent estimates reporting an incidence of up to 1 in 2100 [5], whereas the incidence of DM2 appears to vary considerably across populations [6–8]. Both forms of DM show high penetrance levels [9–11], with > 50 CTG repeats and > 75 CCTG repeats marking the pathological expansion thresholds for DM1 and DM2, respectively [9, 10, 12]. Repeat length shows an inverse relationship with age of onset and a positive correlation to disease severity in DM1 [13–15], due to which four distinct clinical subtypes have been established: congenital, juvenile, adult, and late-onset [16]. This relationship is not observed in DM2, in which affected individuals have disease alleles ranging from 75 to 11,000 repeat units without an apparent effect of expansion length on phenotypic severity or symptom onset [9].

**Fig. 1:**
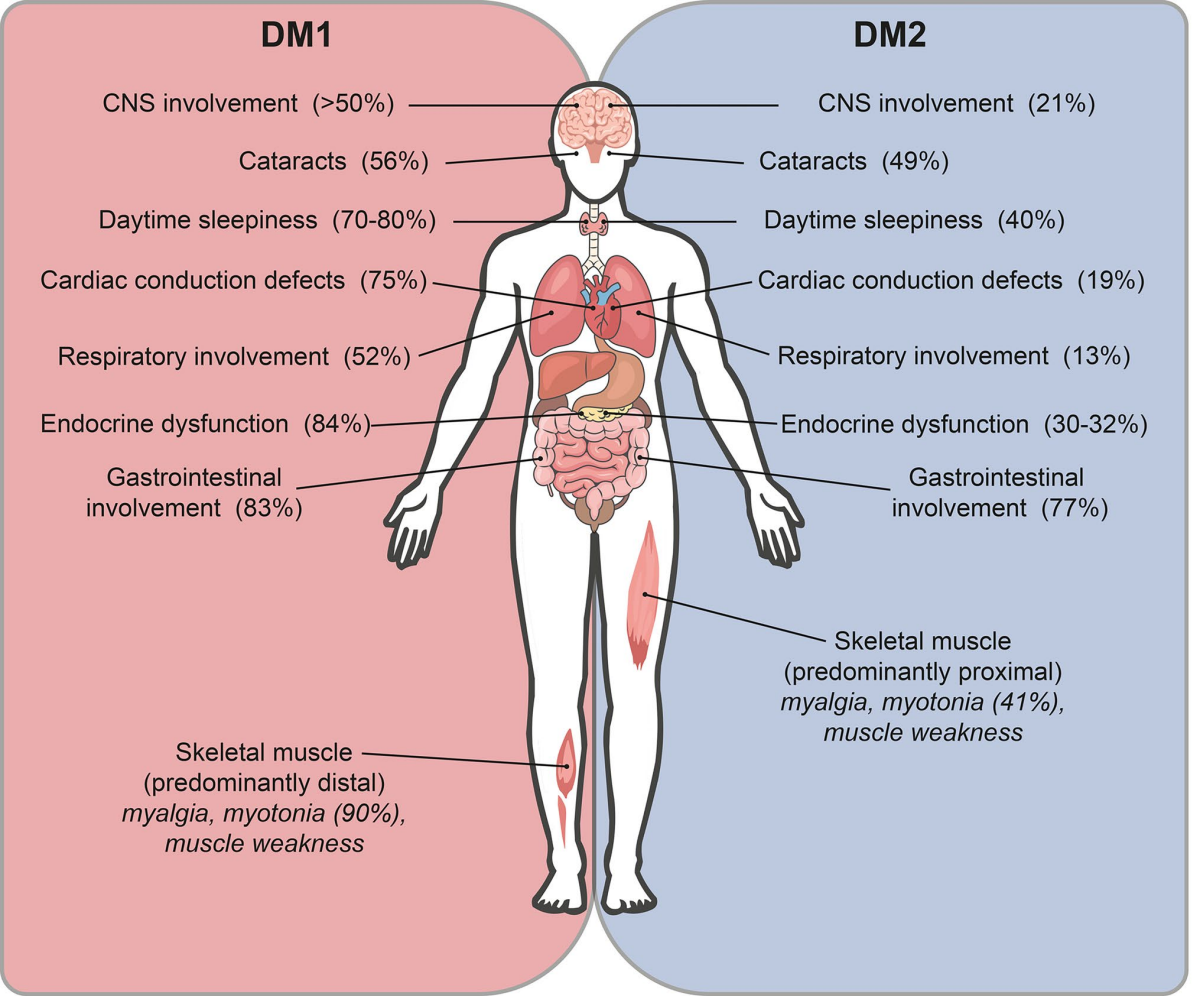
Overview of common manifestations in myotonic dystrophy type 1 (DM1) and type 2 (DM2). As myotonic dystrophies are multisystemic, they present with a broad phenotypic spectrum. There is substantial overlap in clinical manifestations between DM1 and DM2; however, their frequency and severity differ between the two disease forms. Symptom frequencies derived from [6, 10, 24–32]

Aside from hallmark symptoms of myotonia and progressive muscle weakness, DM patients frequently develop cataracts and insulin resistance. Other common manifestations include hypogonadism and involvement of the respiratory, cardiac, central nervous, and gastrointestinal systems (Fig. 1) [9, 17, 18]. Despite substantial phenotypic overlap, DM1 and DM2 exhibit several distinct features, likely reflecting underlying mechanistic differences. DM1 predominantly affects distal musculature and exhibits more pronounced myotonia, frequently involving hand and facial muscles and progressing to bulbar dysfunction and respiratory insufficiency in advanced disease. In contrast, DM2 is similarly characterized by muscle atrophy and chronic myalgia but preferentially affects proximal muscles, with patients exhibiting less myotonia and generally milder bulbar and respiratory involvement [10, 19, 20].

Central nervous system (CNS) involvement represents an important component of disease burden, particularly in DM1, where widespread white matter abnormalities, cortical atrophy, and disruption of fronto-temporal networks contribute to executive and visuospatial dysfunction. While DM2 also demonstrates structural brain involvement, this typically presents as milder and less specific fronto-parieto-occipital changes without the characteristic temporo-insular pattern observed in DM1, and clinically manifests as subtle executive dysfunction, slowed information processing, fatigue, and affective symptoms [21, 22].

In DM1, age of onset and symptom severity is highly variable and closely associated with clinical subtype, with congenital patients typically exhibiting the highest clinical burden [16]. By contrast, DM2 typically manifests in the third-to-fifth decade of life [10, 23]. Additionally, DM2 is not subject to genetic anticipation, which has no obvious congenital form and generally presents with a milder phenotype [9], highlighting key clinical and biological distinctions from DM1.

## Molecular pathology of DM

The partial phenotypic overlap between DM1 and DM2 is attributed to shared underlying molecular pathomechanisms, in particular splicing pathology, referred to as “spliceopathy”. This concept describes the global dysregulation of alternative splicing, often resulting in fetal or truncated, nonproductive transcript isoforms being expressed in adult tissues, with numerous DM manifestations having been linked to specific aberrant splicing events [33–37].

**Fig. 2:**
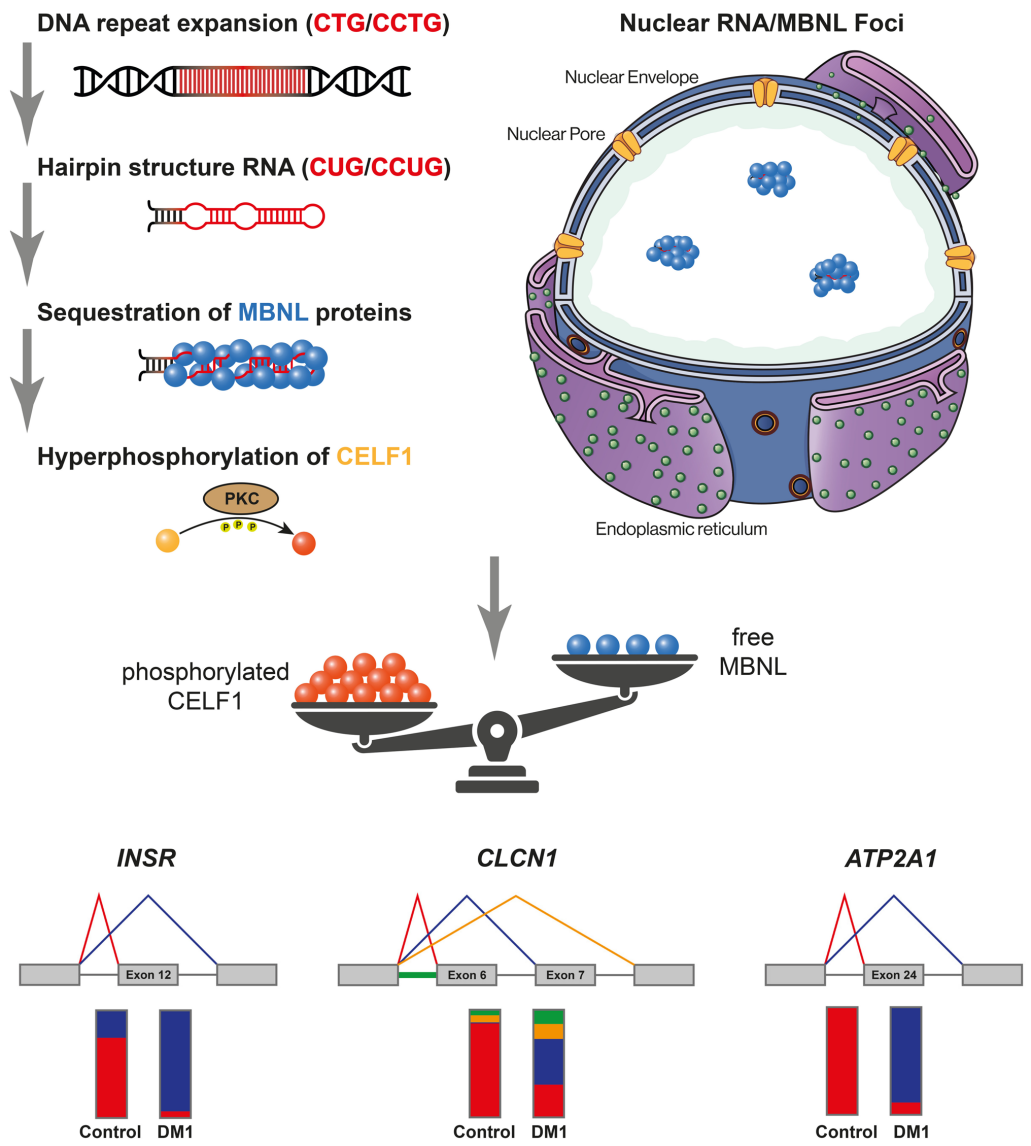
Overview of molecular pathology underlying myotonic dystrophies. Myotonic dystrophy type 1 (DM1) and type 2 (DM2) are caused by CTG and CCTG DNA repeat expansions, respectively. Transcribed pathological repeat expansions form stable RNA secondary structures due to complementarity between cytosine and guanine bases, generating hairpin loops. These hairpin loops remain in the nucleus and sequester muscleblind-like (MBNL) proteins through their affinity for YGCY sequence motifs, leading to the formation of RNA foci and reduced levels of bioavailable MBNL. Simultaneously, the presence of RNA foci is associated with increased protein kinase C (PKC) activity which stimulates hyperphosphorylation of CUGBP Elav-like family member 1 (CELF1). The resulting imbalance between reduced MBNL availability and increased CELF1 activity leads to global disruption of alternative splicing regulation [41–44]. Aberrant splicing patterns observed in DM1 patient biopsies are illustrated for insulin receptor (INSR)*,* chloride voltage-gated channel 1 (CLCN1), and *A*TPase sarcoplasmic/endoplasmic reticulum Ca^2+^ transporting 1 (ATP2A1), highlighting dynamic changes in the proportions of different splice events (single intron excision = red; single exon skipping = blue; double exon skipping = yellow; intron retention = green)

This dysregulation is caused by the formation of abnormal secondary structures of CUG/CCUG repeat RNA, to which muscleblind-like splicing regulator 1 (MBNL1) and its paralogs bind and become sequestered, leading to the formation of nuclear RNA foci (Fig. 2) [3, 38–40]. In parallel, the presence of RNA foci promotes protein kinase C activity, leading to hyperphosphorylation of CUGBP Elav-like family member 1 (CELF1) which increases its stability and activity [41–43].

The resulting imbalance between reduced bioavailable MBNL1 and increased CELF1 activity is thought to disrupt multiple post-transcriptional processes, including alternative splicing, mRNA stability, transport, and translation [33]. Importantly, these molecular alterations form the basis for several biomarker strategies, particularly those targeting aberrant splicing patterns in affected tissues.

However, unlike in DM1, CELF1 hyperphosphorylation has not been consistently observed in DM2 [10]. Analysis of RNA foci suggests MBNL1 sequestration to be more apparent in DM1, while in DM2, MBNL1 appears to compete with other splice proteins, such as RNA-binding Fox-1 (rbFOX) homologs 1 and 2 [10, 45]. Dysregulation of MBNL1, CELF1, and rbFOX1 independently have been associated with aberrant splicing [12, 46–50]. Therefore, while the overall mechanism of altered splicing appears to be shared, ongoing research is beginning to elucidate molecular differences that likely account for some of the aforementioned phenotypic discrepancies between the two diseases. These differences may also influence the performance and specificity of splicing-based biomarkers across DM subtypes.

Other possible disease mechanisms have been proposed for both DM1 and DM2, including haploinsufficiency caused by the heterozygous loss of DMPK and CNBP, respectively. Consistent with this, heterozygous loss of CNBP appears to contribute to the muscle atrophy typical of the DM2 phenotype [51–53]. However, in DM1, evidence suggests that loss of DMPK itself does not drive phenotypic manifestations [54, 55].

Similarly, investigations into the potential influence of changes in the chromatin environment and gene expression on DM pathophysiology have yielded divergent results in DM1 and DM2. In DM1, altered DNA methylation status upstream and downstream of the CTG repeat expansion is consistently observed [56–61] and has been associated with increased repeat instability and aberrant gene expression patterns in consequence of disruption of topological associated domains [62–65]. Conversely, available evidence so far has not detected altered methylation status nor impact on CNBP expression in DM2 [65, 66].

Thus, while shared splicing alterations explain much of the phenotypic overlap, differences in the proteins sequestered by RNA foci in DM1 and DM2, together with the outlined divergent mechanistic insights, are likely to contribute to differences in disease progression and may represent an additional source of biomarker heterogeneity between the two conditions.

## Disease diagnosis and management

Diagnosis of DM1 and DM2 is challenged by the variation in age of onset and a broad phenotypic spectrum, resulting in an average diagnostic delay of approximately 7 and 14 years for DM1 and DM2, respectively [24, 67, 68]. The standard diagnostic approach includes assessment of family history and molecular genetic testing of blood leukocytes, typically using conventional PCR, followed by repeat-primed PCR and Southern blotting if only a single normal-sized allele is detected [10, 69]. Alternative procedures involving fluorescence in situ hybridization and long-read genome sequencing have been developed for diagnosis but are not yet widely implemented in routine clinical practice [70, 71].

Treatment of DM remains primarily symptomatic, as no curative therapy is currently available for either disease form [34, 67]. However, advances in the understanding of underlying molecular mechanisms have led to the development of targeted therapeutic strategies, including antisense oligonucleotides (ASOs) and gene therapies, which are currently in various stages of clinical development [55, 72].

Therefore, both diagnostic delay and increasing emergence of potential treatments, together with the multisystemic and slow progressive nature of DM, highlight the need for sensitive and reproducible biomarkers to recognize and monitor disease progression, as well as to evaluate treatment efficacy.

## Biomarkers for DM

Biomarkers serve as crucial tools in both research and clinical environments, used in diagnosis, prognosis, disease monitoring, and therapy evaluation [73–75]. Key considerations include disease specificity, sampling method, and the associated patient burden, as well as the ability to standardize measurement and interpretation [74].

In the context of DM, repeat length is currently the only biomarker that shows some predictive value for age of onset and disease severity, a relationship that applies exclusively to DM1. However, the strength of this association remains debated, as substantial overlap in repeat length across clinical subtypes limits the definition of clear thresholds [14, 15, 76]. Additionally, variants within the repeat motif, such as repetitive repeat interruptions, appear to have protective effects, making interpretation of repeat length as a biomarker more complex [13].

Importantly, no reliable relationship between repeat length and clinical phenotype has been established in DM2, underscoring the limited availability of validated biomarkers for both disorders and highlighting a particular unmet need in DM2.

## Imaging-based biomarkers

Magnetic resonance imaging (MRI) is a non-invasive technique that provides detailed structural and functional assessment of tissue, with important clinical and research applications. In DM, MRI offers a valuable approach to assess disease involvement and progression, and several studies have proposed standardized protocols for the use of MRI-derived measures as biomarkers.

### Central nervous system MRIs

Given the frequent development of CNS complications in both DM1 and DM2, brain imaging studies have demonstrated that CNS involvement follows partially reproducible patterns, particularly in DM1, where diffuse white matter abnormalities frequently include a characteristic temporo-insular distribution not typically observed in DM2. These structural changes extend beyond focal lesions and involve widespread cortical and subcortical networks, supporting the concept of diffuse rather than regionally confined brain involvement.

Structural MRI studies consistently report gray and white matter atrophy associated with cognitive and behavioral manifestations, as well as altered functional connectivity [21, 77–79]. However, evidence supporting the use of brain MRI as a biomarker remains limited. This includes an early study which failed to correlate brain parenchymal fraction to clinical parameters [80] and the proposition of high-resolution 3 T brain MRIs as a potential biomarker of CNS defects, such as cognitive impairment, depression, and daytime sleepiness, which lacks longitudinal data [77, 81].

While these findings suggest potential utility, current results are inconsistent and insufficient to support standardized clinical implementation of MRI-based biomarkers. Advanced quantitative imaging approaches further suggest that both gray and white matter are globally affected, especially in DM1, although correlations with clinical parameters remain variable across studies. Additionally, the lack of longitudinal data currently limits the validation of MRI-derived measures as reliable markers of disease progression.

In clinical practice, MRI currently serves primarily as a supportive tool to characterize CNS involvement rather than a validated biomarker for disease monitoring. Circulatory biomarkers of CNS involvement have been proposed, such as serum-derived Neurofilament Light Chain (NfL), with MRI scans used as a promising supplementary tool to validate experimental findings [82, 83], suggesting an important adjunctive role for imaging in multimodal biomarker strategies.

### Muscle MRIs

The use of muscle MRIs to evaluate muscle health has also been proposed [84–86]. Studied in DM1, MRI-derived T1 sequences and short-tau inversion recovery (STIR) scores, interpreted from imaging wavelengths, showed strong correlations with the muscle impairment rating scale, muscle atrophy, hypersensitivity, and fat replacement of muscle tissue [84], suggesting their potential value as biomarkers.

Longitudinal assessment demonstrated that altered STIR scores preceded fat replacement, and T1 sequences allowed detection of atrophy and fat replacement prior to noticeable clinical decline [85]. However, the reliability of these findings is limited by small cohort sizes and incomplete longitudinal follow-up.

Alternatively, quantitative MRI (qMRI) of DM1 patients’ forearms showed significant correlation between muscle fat fraction (MFF) and T2 relaxation time with clinical parameters of strength, functional ability, and myotonia [87]. In line with DM1 investigations, a DM2-focused study proposed using MFF and contractile volume (CV) scores as biomarkers. MFF scores inversely correlated, and CV positively correlated with clinical assessments of muscle function [86]. Similarly, Kokosova et al*.* [88] used qMRIs to study the lumbar paraspinal muscles of DM2 patients and correlated the derived MFF scores with muscle strength and endurance.

Despite these associations, relatively low correlation coefficients and limited cohort sizes currently restrict the use of qMRI-derived measures as reliable functional biomarkers. Furthermore, biomarkers evaluated in only one disease subtype require independent validation in the respective other cohort to establish their generalizability. More broadly, the reliability of MRI-based studies is frequently constrained by small sample sizes and a lack of longitudinal data, limiting their prognostic value.

Overall, muscle MRI provides a non-invasive approach to characterize muscle involvement in DM and shows promise as a biomarker modality. However, high costs, limited standardization, and insufficient longitudinal validation currently preclude its routine use as a clinical biomarker, and further large-scale studies are required.

## Muscle-derived biomarkers

As skeletal muscle is the predominantly affected tissue in DM, muscle biopsies represent a key source of pathological information and biomarker assessment, enabling evaluation of both morphological alterations and molecular signatures associated with disease pathology. However, considering muscle biopsy is not routinely required for DM diagnosis, standardized implementation of muscle-derived biomarkers is limited, particularly given the associated patient burden.

### Splicing biomarkers

Muscle samples provide essential insight into the altered splicing underlying DM1 and DM2, with hundreds of genes subjected to aberrant alternative splicing in skeletal muscle [89, 90]. Several specific splice events are consistently observed and have been linked to phenotypic manifestations of DM. These include mis-splicing of insulin receptor (INSR), chloride voltage-gated channel 1 (CLCN1), ATPase sarcoplasmic/endoplasmic reticulum Ca2 + transporting 1 (ATP2A1), bridging integrator 1 (BIN1), sodium voltage-gated channel alpha subunit 5 (SCN5A), and troponin T2 cardiac type (TNNT2), associated with symptoms, such as insulin resistance, myotonia, muscle weakness, and cardiac arrhythmia [89, 91–95].

However, due to the multisystemic nature and variable expressivity of DM phenotype, individual splice events are insufficient as standalone biomarkers to comprehensively reflect disease severity or progression.

Based on the observation that loss of MBNL affects splicing of different genes to variable extents [89, 96–98], MBNL levels have been proposed as a potential biomarker. Intracellular, bioavailable MBNL concentration in biopsied samples showed a strong relationship with muscle strength across different subtypes of DM1, suggesting utility as a marker of disease state [99]. However, the investigation exclusively included DM1 samples and did not account for other possible molecular mechanisms underlying DM1 or DM2.

More recently, a splice index (SI) comprising a panel of 22 splice events was generated for DM1 [100, 101], development of which was encouraged by the growing therapeutic pipeline, underscoring the need for representative biomarkers to evaluate clinical efficacy. The DM1 SI, increasingly referred to as composite alternative splicing index (CASI), showed a moderate relationship with myotonia and a strong correlation with various clinical measures of muscle strength and motor function. Longitudinal analysis spanning a 3-month period detected changes in the SI, during which phenotypic correlations remained robust [101].

While promising, the relationship between SI scores and specific DM1 subtypes remains to be fully defined, and current longitudinal data are limited to short observation periods, restricting assessment of long-term disease progression. Further validation in larger and more diverse cohorts, with extended follow-up, will be required to establish clinical utility, particularly in the context of therapeutic trials (e.g., ACHIEVE: NCT05481879 [102]; MARINA: NCT05027269 [103]) [104–106].

Considering the shared pathomechanism of MBNL1 sequestration, almost identical aberrant alternative splicing profiles would be expected for DM1 and DM2. However, while there is extensive overlap in alternative splicing signatures [107], direct comparison of alternative splicing events reported for DM1 [108] and DM2 [90] also reveals several differences in splicing profiles. These are likely linked to the differences in proteins sequestered to RNA foci in DM1 versus DM2, as MBNL1 competes with proteins like rbFOX1 in DM2 [10, 45]. Furthermore, the splicing mechanism is affected by more than just the sequestration and consequent loss of function on MBNL proteins—in DM1, differential expression of several genes encoding splicing proteins has been reported [109], a feature (including altered splicing) that is shared with different myopathies [108].

Consequently, the DM1-derived SI, developed using tibialis anterior muscle biopsies, is not directly transferable to DM2. The development of DM2-specific splicing biomarkers, likely requiring analysis of proximal muscle groups, remains an important unmet need.

Collectively, splicing biomarkers most closely reflect the pathological mechanism underlying DM, with panels offering a more comprehensive assessment of the phenotype than later-discussed symptom-specific alternatives. However, their longitudinal use is constrained by the invasive sampling nature.

### Proteomic biomarkers

While research commonly focuses on transcriptomic data indicative of altered splicing, proteomic signatures derived from muscle samples for both DM1 and DM2 have also been studied. A 12-week evaluation of the clinical efficacy of strength training in DM1 patients reported significant changes in the expression of 44 proteins involved in immunity, energy metabolism, apoptosis, insulin signaling, myogenesis, and muscle contraction [110], consistent with typical DM manifestations. These findings highlight the potential of proteomic signatures as dynamic biomarkers to monitor therapeutic interventions, although comparable data in DM2 remain limited and require further investigation.

Beyond global proteomic profiling, some investigations focus on finding proteomic biomarkers by studying specific cellular functions known to be altered in DM, such as the hyperactivation of autophagic response observed in DM1 [111–113]. Proteomic analysis focusing on autophagy-related proteins identified death-associated kinase 1 (DAPK1), a regulator of cell death and autophagy [114–116], as significantly upregulated in DM1 patients [117]. Expression levels were inversely correlated with phenotypic severity, implicating promise as a prognostic biomarker. However, because the role of DAPK1 in DM1 pathology is currently unclear, further analysis is needed before its use as a biomarker is established.

Another potential proteomic biomarker is nesprin-1 (nuclear envelope spectrin repeat protein 1), a nuclear envelope transmembrane protein (NET). NETs have been associated with different myopathies [118], including significant downregulation of nesprin-1 and aberrant nuclear envelope morphology reported in DM1 myoblasts [119, 120] as well as altered expression of NETs involved in genome organization in DM1 muscle [121]. While NET biomarkers do not appear to be disease-specific, further investigation into the relationship between nesprin-1 and DM1 pathology may be worthwhile for their possible use as indicators of muscle cell health. Potential uses of DAPK1 and nesprin-1 as biomarkers for DM2 are plausible, based on shared molecular and phenotypic features.

Proteomic analysis of DM2 myotubes identified two major functional categories in which differential expression was observed relative to non-disease controls: mitochondrial components (EFtu, HSP60, GRP75, dienoyl-CoA-isomerase) and the ubiquitin proteasome system (26S proteasome regulatory subunit 13, alpha-6 subunit, RAD23B) [122]. This presents possible proteomic signatures which could be used as DM2 biomarkers upon further investigation, ideally including disease controls to evaluate specificity and longitudinal analysis to increase prognostic reliability. In agreement with the detected differential expression of mitochondrial components, recent investigations have consistently observed downregulation of proteins in the respiratory chain complexes I, III, and IV (mt-CO1, mt-ND5, mt-CYB, SLC41A3) and translational activator of cytochrome c oxidase I (TACO1). Additionally, morphological signs of mitochondrial dysfunction were found, including colocalization of mitochondrial and autophagic markers in DM2 muscle samples [123].

Similar mitochondrial alterations have also been described in DM1, including reduced levels of coenzyme Q10 (Coq10) [124], although these changes may be more pronounced in DM2, suggesting potential for differential biomarker development between the two disease forms.

Finally, repeat-associated non-AUG (RAN) translation has been reported for both DM1 and DM2, presenting the possibility of developing RAN peptide biomarkers. Investigation in muscle and fibroblast cell cultures detected RAN peptides from the antisense strand (poly-glutamine) in DM1 and both sense and antisense strands (LPAC and QAGR) in DM2 [125–127]. Coherent with RAN translation being implicated in different microsatellite neurodegenerative diseases [128–130], involvement of RAN translation in CNS pathology of DM has been hypothesized following detection of RAN peptides in DM2 patient brains [127].

While RAN peptides confer a relatively high level of disease specificity, their biomarker potential is constrained by limited detection ability in the currently used assays, particularly in DM1 [126]. Nevertheless, RAN protein levels as potential biomarkers in DM1 and DM2 need to be further explored.

Overall, several proteomic biomarkers reflecting aspects of DM pathology have been proposed, but their routine clinical use has yet to be established in DM1 and DM2.

### Non-coding RNA biomarkers

MicroRNAs (miRNAs) are small, non-coding RNA fragments that modulate the expression of specific target genes as a result of complementary sequence affinity [131, 132]. Dysregulation of miRNA levels has been reported across multiple neuromuscular disorders [133, 134].

In DM1, analysis of muscle biopsies found significantly altered levels of miR-1, miR-29b/c, miR-33, and miR-335, as well as abnormal cellular distribution of miR-1, miR-133b, and miR-206 in skeletal muscle (Table 1). Functional relevance was inferred from significantly altered expression of the miRNA target genes [135]. Similar results have been reported for the less well-researched DM2 profiles, including altered levels of miR-34a-5p, miR-34b-3p, miR-125b-5p, miR-146b-5p, miR-193a-3p, miR-208a, miR-221-3p, miR-378a-3p, and miR-381 with pathological consequences inferred from validated miRNA–mRNA interactions [136].

**Table 1:** Overview of miRNAs evaluated in DM1 and DM2 skeletal muscle and serum

miRNA	Studies reporting dysregulation				References
	DM1		DM2		
	Muscle	Serum	Muscle	Serum	
miR-1	↑ (1/2)	↑ (3/3)	↑ (1/1)	↑ (1/1)	[135–137, 146–148]
miR-23b	↑ (1/1)	0	0	0	[141]
miR-24-3p	0	↑ (1/1)	0	0	[149]
miR-27b	0	↑ (1/1)	0	↔ (0/1)	[146]
miR-29b/c	↓ (1/1)	0	0	0	[135]
miR-33	↓ (1/1)	0	0	0	[135]
miR-34a/b/c-5p	↔ (0/1)	0	↓ (1/1)	0	[135, 136]
miR-125b-5p	↔ (0/1)	0	↑ (1/1)	0	[136]
miR-133a/b	0	↑ (3/3)	0	↑ (1/1)	[146–148]
miR-140-3p	0	↑ (1/1)	0	↑ (1/1)	[146]
miR-146b-5p	↔ (0/1)	0	↓ (1/1)	0	[136]
miR-193a-3p	↔ (0/1)	0	↑ (1/1)	0	[136]
miR-193b-3p	↑ (1/1)	0	↑ (1/1)	0	[136]
miR-221-3p	↔ (0/1)	0	↓ (1/1)	0	[136]
miR-206	↑ (1/2)	↑ (3/3)	↔ (0/1)	↑ (1/1)	[135–137, 146–148]
miR-208a	↓ (1/1)	0	↓ (1/1)	0	[136]
miR-218	↑ (2/2)	0	0	0	[141, 143]
miR-223-3p	0	↑ (1/1)	0	0	[149]
miR-335	↑ (1/1)	0	0	0	[135]
miR-378a-3p	↔ (0/1)	0	↑ (1/1)	0	[136]
miR-381	↓ (1/1)	0	↓ (1/1)	0	[136]
miR-454	0	↑ (1/1)	0	↑1/1	[146]
miR-574	0	↑ (1/1)	0	↑1/1	[146]

Detection of significantly altered miRNAs in skeletal muscle and serum of myotonic dystrophy type 1 (DM1) and type 2 (DM2) is indicated by arrows (upregulation by ↑, downregulation by ↓, no significant change by ↔)

The values denote the number of studies in which miRNA levels were found to be significantly altered relative to the number of studies which evaluated the miRNA

Reference numbers correspond to the original publications

However, detection of changes in skeletal muscle-specific miRNAs (myomiRNAs) levels is inconsistent, with exclusively miR-206 appearing significantly altered in an investigation by Gambardella et al*.* [137], while a *Drosophila melanogaster* DM1 model showed a transcriptomic profile involving differential expression of 19 myomiRNAs [111]. Additionally, while some functional relationships between specific miRNAs and DM pathology have been discovered, such as the recently demonstrated relationship between DM1-related miR-107 reduction promoting activity of the atrophy-associated MSI2-MiR-7-autophagy axis [138], other evidence fails to establish a connection between the level of miRNA dysregulation and skeletal muscle health [139]. Taken together, variability between studies and limited mechanistic understanding currently limit the use of muscle-derived miRNAs as robust biomarkers in DM.

Despite these challenges, investigation of miRNAs remains warranted, as emphasized by accumulating evidence supporting the relevance of studying and targeting miR-23b and miR-218 in the context of DM. Significant upregulation of the two miRNAs was reported in data from DM1 muscle biopsies and patient-derived myoblast cell cultures [140, 141], with DM1-related elevation further validated in mouse models [140, 142]. Functional characterization has shown that miR-23b and miR-218 act as repressors of MBNL expression [140, 142], encouraging their investigation as potential treatment targets. Silencing of miR-23b and miR-218 was found to partially rescue dysregulated alternative splicing and improve functional phenotype in DM1 myoblasts and DM1 mouse models [140, 141, 143].

Building on this, recent preclinical evidence has shown great promise of ASO-mediated decrease of miR-23b overexpression in DM1 promoting correction of aberrant alternative splicing events and ameliorating functional phenotype without evidence of toxicity or immune response concerns [144]. Consistent with these findings, inhibition of miR-23b expression via ASO technology is currently in a phase I/II clinical trial (ArthemiR: NCT06300307 [145]) in DM1 patients.

These findings highlight the potential of miRNAs as both mechanistically informative biomarkers and therapeutic targets. However, further validation in larger patient cohorts, improved reproducibility across studies, and extension of analyses to DM2 are required before clinical application can be established.

A relatively recent discovery was the association of dysregulated levels of circular RNAs (circRNAs) with DM1 [150–153]. Like miRNAs, circRNAs are non-coding and appear to show tissue-specific expression patterns, thereby representing potential biomarker candidates [154–156]. CircRNAs are thought to regulate gene expression by interacting with other RNA species and modulating transcription, translation, and mRNA stability, often through miRNA sponging mechanisms [154, 156–158].

Analysis of skeletal muscle biopsies from DM1 patients identified significant upregulation of circARHGAP10, which showed a positive relationship with phenotypic severity and CTG length. Reversing the upregulation of circARHGAP10 decreased MBNL1 sequestration in cell culture. Increased MBNL1 bioavailability was attributed to the interaction of circARGHAP10 with miR-409-3p, specifically demonstrating that amelioration of upregulated circARHGAP10 coincides with a decrease in miR-409-3p levels [159].

These findings indicate that circARHGAP10 may serve both as a mechanistically informative biomarker and a potential therapeutic target in DM1. However, validation in larger patient cohorts is required, and its relevance in DM2 remains to be determined. Notably, circRNA upregulation has also been detected in DM1 blood samples [153], suggesting that circARHGAP10 may have potential as a minimally invasive circulating biomarker.

Non-coding biomarkers represent a promising area for biomarker development and therapeutic target identification; however, many of the discussed markers require further characterization to clarify their role in DM pathology and phenotypic impact before routine implementation.

## Soluble biomarkers

Despite the demonstrated promise of several muscle-derived biomarkers, the high patient burden of muscle biopsies limits their longitudinal application, particularly in the context of disease monitoring and clinical trials. Consequently, recent research has shifted toward developing minimally or non-invasive alternatives, particularly soluble biomarkers [160].

### Proteomic markers of muscle damage

The predominant and only clinically established circulatory biomarker for DM is creatine kinase (CK) [161, 162], a marker of muscle damage also measured in other muscular dystrophies, such as Duchenne muscular dystrophy [163–165].

Similar to serum CK, the potential of using urine samples to measure titin:creatinine ratios as indicators of muscle health was investigated in DM1 patients [166]. Titin degradation occurs in response to injury and urinary titin has been associated with both acute and chronic skeletal muscle damage [167–169].

Elevated urinary titin:creatinine ratios were able to distinguish DM1 patients from healthy controls [166], suggesting potential use as a biomarker of muscle damage. While the information provided is broadly comparable to that of serum CK, the non-invasive nature of urine sampling represents a practical advantage for repeated measurements and longitudinal monitoring.

Another potential biomarker of skeletal muscle health, specifically muscle weakness, is glycogen synthase kinase 3 beta (GSK3ß). Measured in the serum of DM1 patients, including different disease subtypes (congenital, juvenile, and adult), active GSK3ß was elevated in DM1 patients compared to healthy controls and showed a positive correlation to both repeat length and clinical parameters of muscle weakness. Within the same investigation, transforming growth factor-ß (TGFß) was tested as a potential biomarker of muscle health and fibrosis, but results lacked consistency across the different DM1 subtypes [170].

Disease specificity of these markers is likely limited, given that inhibition of GSK3ß is a treatment target in development for various neurodegenerative and muscular disorders [171–173]. Nevertheless, GSK3ß may present a more consistently elevated biomarker, suitable for all subtypes of DM1, compared to CK levels, which can appear normal in DM1 patients [161]. While promising preliminary results were presented, the evaluation was based on the observation of consistent elevation in a relatively small patient cohort [170], and the comprehensive interpretation of the value of GSK3ß as a monitoring biomarker is limited by the lack of longitudinal data.

In summary, several minimally or non-invasive alternative markers of muscle damage have been proposed but have yet to be implemented as supplementary readouts or replacements for CK in clinical routine.

### Symptom-specific biomarkers

Given the multisystemic nature of DM, biomarkers reflecting different phenotypic manifestations may be useful for monitoring symptoms and assessing treatments. Examples include periostin, NfL, total tau (T-tau), phosphorylated tau (P-tau), and beta-amyloid 42 (A*ß*_42_), which represent potential biomarkers of cardiac and CNS defects [174, 175].

Periostin plays a role in cardiac tissue remodeling during normal development, as well as in response to cardiac injury [176–178], therefore suggesting an opportunity for use as a marker of cardiovascular stress and fibrosis in cardiac and skeletal tissue. Following the detection of significantly lower periostin levels in the sera of DM1 patients compared to those of DM2 patients and healthy controls, periostin was suggested as a potential biomarker for DM1 patients considered at risk of cardiac pathology. This was supported by periostin decrease demonstrating an inverse relationship with CTG repeat length, clinical severity and cardiac dysfunction in DM1 [174].

Also studied in DM1 patients was NfL as a biomarker of CNS involvement. Elevated NfL levels in patient sera were consistently detected [82, 179]; however, significant correlation with clinical assessments of cognitive ability was observed for only one of multiple tests [82], thus limiting the breadth of information interpretable from this biomarker. While DM2 patients can also manifest CNS-related symptoms, severity and frequency are typically reduced, indicating that the use of NfL as a potential biomarker may be less suitable.

Similarly, potential use of T-tau, P-tau, and A*ß*_42_ as biomarkers in cerebrospinal fluid (CSF) was tested in juvenile and adult DM1 patients, but no consistent correlation with cognitive testing parameters was observed [180].

In a broader investigative approach, proteomic profiling of CSF from DM1 patients revealed several proteins that were differentially expressed compared to healthy controls. Pathway analysis and theoretical interpretation supported the role of changes in candidate protein levels in contributing to CNS pathology, given their known functions in neuronal health, neuroinflammation, neuromuscular junction integrity, and cytoskeletal regulation [181]. Thus, these preliminary findings have identified candidate proteins with CNS biomarker potential; however, small sample sizes, limited male representation, and the lack of correlational analysis with CNS symptoms constitute limitations that future investigations will need to address. Additionally, CSF is typically sampled via a lumbar puncture, which patients often find uncomfortable, though it remains less invasive than muscle biopsies.

Overall, multiple soluble biomarkers have shown potential for symptom-specific monitoring of DM1, whereas suitable biomarkers for DM2 remain comparatively limited.

### Soluble splicing biomarkers

In contrast to symptom-specific biomarkers, efforts to capture disease pathology more comprehensively through soluble biomarkers are under development. Analogous to the muscle-derived DM1 splice index (SI), Antoury et al. [182] attempted to generate a composite splicing biomarker measured in urine. The primary focus was on DM1 patients; however, DM2 samples were also included. Analysis revealed splicing signatures in urine that resembled those observed in muscle tissue. However, the lack of correlation between urinary splicing profiles and clinical muscle phenotype has limited their utility as reliable biomarkers.

### MyomiRNAs in serum

Altered serum myomiRNAs levels have been detected in both DM1 and DM2 patients, providing a minimally invasive alternative to muscle-derived measures of miRNAs (Table 1). In an investigation of DM1 patients and a DM mouse model, miR-223-3p and miR-24-3p were identified as potential serum biomarkers, with target analysis implicating their role in neuronal and muscle development. Although miR-223-3p may be involved in DM1 pathology, its direct relationship to clinical phenotype has not been systematically evaluated [149].

Analysis of a panel of 12 myomiRNAs in the serum of DM1 and DM2 patients identified eight (miR-1, miR-27b, miR-133a, miR-133b, miR-140-3p, miR-206, miR-454, miR-574) as dysregulated in a manner recapitulating initial observations reported in skeletal muscle and found a weak but significant correlation to both muscle strength and CK levels [146]. Similarly, another investigation observed significant elevations in four myomiRNAs (miR-1, miR-133a, miR-133b, miR-206) in DM1 serum and showed strong correlations with progressive muscle wasting, enabling the identification of patients actively experiencing muscle atrophy [147].

Follow-up investigation validated these findings in a larger patient cohort and reported that myomiRNAs in serum are encapsulated within exosomes, suggesting a mechanism by which atrophying muscle tissue leak myomiRNAs into the circulation [148]. While no correlation to DM1 severity status, CTG length, or patient age was established, high specificity in distinguishing DM1 patients experiencing progressive wastage from non-progressive patients was demonstrated, implicating the informative value of the serum myomiRNAs as biomarkers to monitor muscle wastage [147, 148].

To support the establishment of the four myomiRNAs as serum-based biomarkers, additional longitudinal assessment of the relationship between elevated myomiRNAs levels and the rate of muscle degeneration would be beneficial to add prognostic value. These candidate biomarkers should also be evaluated in DM2 cohorts, given muscle atrophy represents a hallmark feature shared with DM1.

Finally, given the consistent upregulation of miR-23b and miR-218 in DM1 muscle [140, 141], analysis of their expression in serum represents a promising avenue for the development of additional circulating biomarkers in DM1 and DM2.

Providing an example of the implementation potential of soluble myomiRNAs, a 3–6-week exercise rehabilitation program tested whether serum myomiRNAs and myostatin supported the evaluation of treatment efficacy. Improved exercise performance, as reflected by exercise parameters, correlated with downregulation of the chosen biomarkers, supporting their potential as indicators of muscle function, although the study was somewhat limited by a small cohort and short duration [183].

Overall, while the described investigations point to the remarkable promise of serum-based myomiRNA biomarkers, simultaneous analysis of miRNAs in serum and skeletal muscle from DM1 patients has revealed notable discrepancies between the two sample types, suggesting that serum may not directly reflect the biological state in muscle [184]. Additionally, consistent detection of changes in myomiRNAs remains challenging, as highlighted by the failure to detect serum changes in myomiRNAs previously reported as dysregulated in prior studies [185].

Furthermore, serum analysis of DM2 patients has only been sparsely covered, with myomiRNA signatures identified by Perfetti et al*.* [146] yet to be recapitulated in follow-up investigations. Therefore, while serum myomiRNAs merit continued investigation to facilitate biomarker development, disease specificity, detection consistency, and pathological implications need to be further elucidated for their use to become clinically established.

### Autoimmunity biomarkers in DM2

An increased prevalence of autoimmune disease has been reported in DM2, but not in DM1 [186–189]. Accordingly, several investigations have explored potential development of autoimmune biomarkers, including analyses of antinuclear antibody elevation, IgG and IgM signatures [19, 188–190].

The larger repeat expansions typical of DM2 have been hypothesized to be the mechanistic link between increased mitochondrial stress and autoimmune disease susceptibility. This was supported by detection of elevated interferon (IFN) activity scores and type-I IFN-stimulated gene signatures in blood coinciding with upregulated autoimmune responses and increased mitochondrial and endoplasmic reticulum stress [190].

Alternatively, or perhaps synergistically, an autoimmunity risk locus (3q13) is located adjacent to CNBP*,* suggesting possible co-segregation of DM2 alleles with autoimmune deficiency [188]. Moreover, the function of CNBP itself has been implicated in immune and inflammatory response [177–179].

Therefore, while the underlying pathology of autoimmune susceptibility in DM2 remains unresolved, the available evidence underscores the value of establishing biomarkers to assess this aspect of phenotype.

## Conclusions

DM1 and DM2 are multisystemic disorders characterized by clinical heterogeneity and shared, but not identical, molecular pathology. The continuously increasing understanding of the mechanisms underlying DM, notably including the RNA-mediated altered splicing, has driven biomarker development to support diagnosis, prognosis, and disease monitoring. This interest is further motivated by the growing therapeutic pipeline, which requires representative biomarkers to assess clinical efficacy.

This review covers the current state of biomarker development in DM1 and DM2, encompassing imaging-based, muscle-derived, and soluble approaches. While muscle-derived biomarkers, particularly those reflecting dysregulation of alternative splicing profile, such as the DM1 SI, provide direct insight into disease mechanisms and capture disease phenotype despite clinical heterogeneity, their reliance on invasive sampling limits longitudinal applicability and patient acceptability.

Imaging-based alternatives have been suggested, particularly for assessing CNS involvement and muscle health, but their development is impeded by high costs limiting accessibility and a lack of standardized protocols. Consequently, recent focus has shifted toward development of soluble biomarkers, which provide minimally or non-invasive alternatives. Particularly, serum myomiRNAs have generated excitement; however, inconsistent detection, questions of disease specificity, and limited longitudinal validation will need to be addressed prior to their implementation in routine clinical use.

The disproportionate focus of research on DM1 highlights the lack of biomarkers explicitly studied for use in DM2, despite scarcity of validated biomarkers applying to both disorders. While shared applicability of several of the discussed biomarkers to DM1 and DM2 is plausible, targeted research is needed for confirmation. Emerging evidence has also highlighted pathophysiological differences between DM1 and DM2, including differing levels of mitochondrial dysfunction and autoimmune susceptibility. This emphasizes the importance of validating biomarker suitability in DM1 and DM2 independently and suggests potential avenues for future biomarker research, which may aid in distinguishing the two disease forms.

Ultimately, advances in biomarker research for DM1 and DM2 will be essential for reducing diagnostic delays, discovering new treatment targets for personalized medicine, and assessing new therapeutic strategies to support their use in clinical practice.

## Data Availability

Data availability is not applicable.
